# Biofilm on Toothbrushes of Children with Cystic Fibrosis: A Potential Source of Lung Re-Infection after Antibiotic Treatment?

**DOI:** 10.3390/ma15062139

**Published:** 2022-03-14

**Authors:** Honghua Hu, Nicole Clothier, Anita Jacombs, Karen Mckay, Anand K. Deva, Karen Vickery

**Affiliations:** 1 Faculty of Medicine, Health and Human Sciences, Macquarie University, Sydney, NSW 2109, Australia; nicole.ev.clothier@gmail.com (N.C.); anita.jacombs@mqhealth.org.au (A.J.); anand.deva@mq.edu.au (A.K.D.); karen.vickery@mq.edu.au (K.V.); 2 Department of Respiratory Medicine, The Children’s Hospital at Westmead, Sydney, NSW 2145, Australia; karen.mckay@health.nsw.gov.au

**Keywords:** cystic fibrosis, recurrent lung infection, antibiotic treatment, biofilm, toothbrush

## Abstract

Frequent recurrent lung infections result in irreversible lung damage in children with cystic fibrosis (CF). This study aimed to determine if toothbrushes contain biofilms of pathogens, and act as potential reservoirs for lung re-infection following antibiotic treatment of acute exacerbations. Toothbrushes were collected from children with CF of lung infection before, during and after antibiotic treatment. Toothbrushes were rinsed with sterile saline and cultured. Bacterial isolates from toothbrushes were identified by 16s rRNA gene sequencing and compared with isolates from a sputum sample of the same patient. Scanning electron microscopy (SEM) was used to visually confirm the presence of bacterial biofilms and confocal laser scanning microscopy (CLSM) combined with Live/Dead stain to confirm bacterial viability. Large numbers of bacteria and biofilms were present on all toothbrushes. SEM confirmed the presence of biofilms and CLSM confirmed bacterial viability on all toothbrushes. Pathogens identified on toothbrushes from children before and during antibiotics treatment were in concordance with the species found in sputum samples. *Pseudomonas aeruginosa* and *Staphylococcus aureus* was able to be cultured from children’s toothbrushes despite antibiotic treatment. Toothbrushes were shown to be contaminated with viable pathogens and biofilms before and during antibiotic treatment and could be a potential source of lung re-infections.

## 1. Introduction

People with Cystic fibrosis (CF) have an impaired ability to clear mucous from the respiratory tract leaving them vulnerable to lung infections [1]. Infections of the respiratory tract are the major cause of morbidity and mortality for people with CF. *Pseudomonas aeruginosa* and *Staphylococcus aureus* are the most common causes of lung infections in children suffering from CF [2]. Early colonization and infection of the lungs is generally dominated by *S. aureus*, but over time, this infection is gradually replaced and dominated by the ubiquitous organism *P. aeruginosa* [3].

Clinically initial CF lung infections are relatively mild and are cleared by aggressive antibiotic treatment [4]. However, development of new and recurrent lung infections is common in children with CF and a cycle of clearance and infection ensues [2]. Although pathogens are eradicated from the lungs between periods of active infections, pulmonary inflammation and structural damage still occur, eventually leading to chronic lung infection. By their teenage years, an excess of 80% of CF patients are chronically infected with *P. aeruginosa* [5]. *P. aeruginosa* chronic lung infection is a major cause of morbidity and mortality in CF patients. However, the source of recurrent infections is unknown. This makes prevention difficult.

*P. aeruginosa* readily forms biofilms in moist environments. A biofilm is a complex community of microbial aggregates that are attached to a surface and to each other by extracellular polymeric substances (EPS). Biofilms can be 100-fold more resistant to antibiotics treatment than planktonic cells and are the basis of many persistent diseases [6].

Environmental contamination is an important reservoir of pathogens in healthcare settings. A study has found that the *P. aeruginosa* isolates from nine infants with CF home environment were the same genotype of the patient’s clinical isolates [7]. Genotyping of longitudinally obtained *P. aeruginosa* clinical isolates from children with CF showed the same genotype in repeated episodes of lung infections [8]. Our previous study also found the same *P. aeruginosa* genotype in clinical samples and in two children’s home environments [8]. Both children were well with negative respiratory specimen cultures for *P. aeruginosa* at the time of home environmental sampling (unpublished information).

Toothbrushes have been reported as a potential environmental reservoir for lower airway infections of children with CF [9] given their daily use in close association with the respiratory tract. We hypothesized that live pathogens can be protected inside biofilms on toothbrushes during antibiotic treatment and could be a potential source for lung re-infection of children with CF after antibiotic treatment. In this study, we evaluated toothbrushes used by children with CF before, during and after antibiotic treatment for the presence of biofilms and live pathogens.

## 2. Materials and Methods

### 2.1. Bacterial Species Identification from Sputum Samples

Human ethics approval was obtained from Sydney Children’s Hospitals Network Human Research Ethics Committee (Approval number LNR/13/SCHN/392). All sputum samples were collected from children with CF during acute exacerbation at the Childrens’ Hospital at Westmead, Sydney, Australia. The Childrens’ Hospital at Westmead routinely treats early acute CF lung *S. aureus* infections with 3 weeks of oral antibiotics and treats early *P. aeruginosa* infections with 4 weeks of inhaled antibiotics in conjunction with 2 weeks of oral antibiotics.

Clinical sputum samples from children with CF were processed using standard routine microbiological techniques by the Microbiology Department, The Childrens’ Hospital at Westmead. Phenotypic identification of culturable isolates was determined using the VITEK 2 system (bioMérieux, Marcy-l’Étoile, France).

### 2.2. Toothbrush Collection and Sample Processing

Toothbrushes were randomly collected from six children with CF aged 8 to 15 with one toothbrush collected from each child. At the time of toothbrush collection, two children had acute infection before antibiotic treatment, two children had been receiving antibiotic treatment for two or three weeks, and two children currently without acute infection, but had received antibiotic treatment in the past and replaced their toothbrushes after starting and finishing their antibiotic treatment (Table 1).

Toothbrushes were rinsed in sterile phosphate-buffered saline (PBS) twice to remove loosely adherent bacteria. Bundles of toothbrush bristles were aseptically pulled out using sterile clamps and subjected to culture for detection of bacteria. The presence of live bacteria, remaining on toothbrush bristles, was confirmed using the LIVE/DEAD^®^ BacLight™ Bacterial Viability Kit (ThermoFisher Scientific, Waltham, MA, USA) and confocal laser scanning microscopy (CLSM) and a biofilm was visually confirmed by scanning electron microscopy (SEM).

### 2.3. Biofilm Bacterial Culture and Identification

Two bundles of toothbrush bristle from the top, middle and bottom areas of each toothbrush were pulled out for study using sterile clamps. Each bundle of toothbrush bristle was placed in 10 mls tryptone soya broth (TSB, ThermoFisher Scientific, Waltham, MA, USA) and sonicated in an ultrasonic bath (Soniclean, Dudley Park, SA, Australia) for 15 min with a sweeping frequency of 42–47 kH at 25 °C. The sonicated solution was subjected to serial dilution and plate culture on horse blood agar, pseudomonas isolation agar and mannitol salt agar. The culture plates were incubated at 37 °C for 24 to 48 h and colony forming units (CFU) were counted.

Bacterial species of isolates with different colony morphology recovered from toothbrush bristles were identified by 16S rRNA gene sequencing according to the method described by Kidd [10].

### 2.4. Confocal Laser Scanning Microscopy

Toothbrush bristles were stained with a LIVE/DEAD^®^ BacLight™ Bacterial Viability Kit (ThermoFisher Scientific, Waltham, MA, USA) according to manufacturer’s instructions. Membrane-permeant SYTO^®^ 9 labels live bacteria with green fluorescence; membrane-impermeant propidium iodide labels membrane-compromised bacteria with red fluorescence. Stained samples were examined using a Fluoview 300 inverted confocal laser scanning microscopy system (Olympus Corporation, Tokyo, Japan).

### 2.5. Scanning Electron Microscopy

Sections of toothbrushes were fixed in 3% glutaraldehyde (Sigma-Aldrich, St. Louis, MO, USA), dehydrated in alcohol prior to immersion in hexamethyldisilazene (Sigma-Aldrich, St. Louis, MO, USA) for 3 min before being aspirated dry and allowed to evaporate in a desiccator overnight. The dried samples were sputter-coated with a 20 nm gold film and examined under a JEOL JSM-6480LA scanning electron microscope (Serving Advanced Technology, Peoria, IL, USA) at Macquarie University Microscopy Unit.

### 2.6. Statistical Analysis

A Mann–Whitney Rank Sum test was used to examine for differences between the total number of biofilm bacteria per bundle of bristles in toothbrushes obtained from children who were receiving antibiotics treatment compared to those before antibiotic treatment using the SigmaPlot 13 statistical program (Systat Software Inc., San Jose, CA, USA).

## 3. Results

### 3.1. Bacterial Species from Sputum Samples

Of the six children with CF, five had *P. aeruginosa* infections; two children were also co-infected with *S. aureus*, one child was co-infected with Haemophilus influenzae. One child (child 6) had a *Stenotrophomonas maltophilia* lung infection only (Table 1).

### 3.2. Bacterial Number and Species from Toothbrushes

Children with acute exacerbations of lung infection had significantly more culturable bacteria (*p* = 0.008) contaminating their toothbrushes than children with their infections under control. The toothbrushes from children with acute exacerbations of disease had over 100-fold cultured bacteria than toothbrush samples obtained from children 2 to 3 weeks following commencement of antibiotic treatment (Table 1). *P. aeruginosa* and *S. aureus* was cultured from the toothbrushes of children with acute lung infections before antibiotics treatment and during antibiotic treatment but was not isolated from toothbrushes of children 3 or 7 months after antibiotic treatment (Table 1). Oral bacterial species were isolated from replaced toothbrushes of children after antibiotic treatment (Table 1).

### 3.3. Confocal Laser Scanning Microscopy

**Figure 1:**
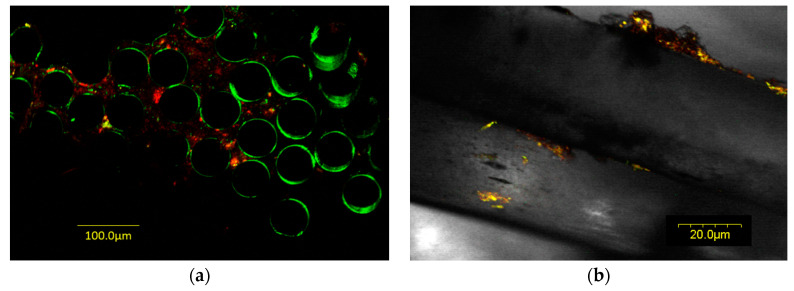
Confocal laser scanning microscopy image of toothbrush bristles with bacteria stained with LIVE/DEAD^®^ BacLight™ Bacterial Viability Kit. Live bacteria are stained green and dead bacteria are stained red. (**a**) Toothbrush from a child with CF before antibiotic treatment; (**b**) toothbrush from a child with CF receiving antibiotic treatment.

Confocal laser scanning microscopy (CLSM) showed that live bacteria were present in biofilms on all toothbrush samples. Figure 1a is an example of a toothbrush collected from a child with CF before antibiotic treatment. Figure 1b is an example of a toothbrush collected from a child with CF receiving antibiotic treatment.

### 3.4. Scanning Electron Microscopy

**Figure 2:**
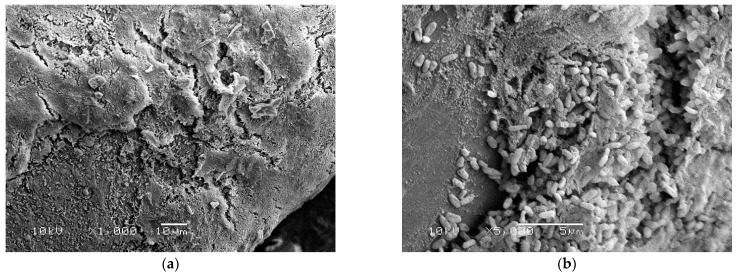
Scanning electron microscopy images of toothbrushes obtained from children with active disease before antibiotic treatment showing large numbers of bacteria both attached to and encased in extracellular polymeric substances (EPS) on toothbrush bristles. (**a**) Low power magnified ×1000; (**b**) high power magnified ×5000.

**Figure 3:**
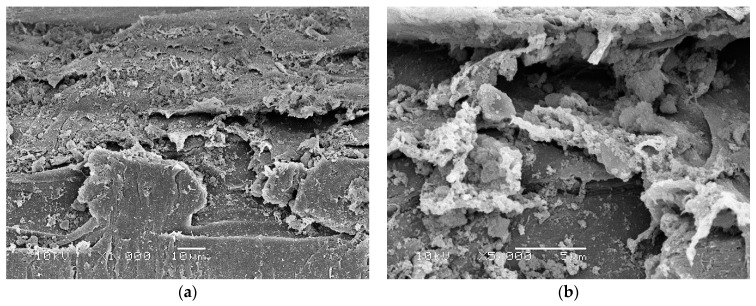
Scanning electron microscopy images of toothbrushes obtained from children receiving antibiotics treatment showing thick EPS with little surface bacteria, suggesting that bacteria are completely encased in thick EPS. (**a**) Low power magnified ×1000; (**b**) high power magnified ×5000.

Scanning electron microscopy (SEM) identified a biofilm with large numbers of bacteria attached to and surrounded by thick EPS on toothbrushes obtained from children with active disease before antibiotic treatment (Figure 2). In contrast, toothbrushes obtained from children receiving antibiotics treatment showed that biofilm bacteria were completely encased inside thick EPS (Figure 3).

## 4. Discussion

We have demonstrated that toothbrushes used by children suffering from CF are contaminated with large quantities of viable bacteria, including *P. aeruginosa* and *S. aureus*, residing inside biofilms before receiving and during antibiotic treatment. Not surprisingly, we also cultured quite a few oral bacteria including *Streptococcus* and *Enterococcus* spp. from toothbrush samples due to contamination by oral flora.

Biofilm bacteria have increased resistance to antibiotics and environmental insults such as desiccation. Within biofilms, bacteria are also more resistant to biocides. This explains the ability of bacteria to form a biofilm on toothbrushes in the presence of triclosan, an active ingredient present in many brands of toothpaste. The number of *P. aeruginosa* contaminating toothbrush bristles was reduced but not eliminated from the children receiving clinically targeted antibiotic treatment.

SEM showed that biofilms on toothbrushes in the absence of antibiotic treatment consisted of large numbers of bacteria both attached to and surrounded by thick EPS (Figure 2). In contrast, in toothbrush samples obtained from children who had received antibiotic treatment, a large amount of EPS is present but obvious surface bacteria were absent suggesting that these superficial bacteria are killed by antibiotics present in the child’s saliva (Figure 3). However, bacteria deeper in the biofilm covered by EPS are protected from antibiotics and remain alive as demonstrated by live/dead staining (Figure 1b) and by culture in the case of children 3 and 4 (Table 1).

A study compared the bacteria isolated from toothbrushes/saliva and sputum samples of 13 adults and 25 children with CF, and found the same bacterial species were present in sputum and saliva samples in 31 out of 38 patients studied; and 10 patients also had the same bacterial species present in their toothbrush contemporary [9]. To verify the possibility of transmission between mouth/toothbrush and lower airways, the authors performed a genotyping analysis of the bacterial isolates present in different sites and found clonal strains present in sputum and saliva/toothbrush samples in almost all cases with only two exceptions [9]. The results suggest that the bacteria in lower air way infections could present in patients’ oral cavity and contaminate their toothbrushes; on the contratry, the toothbrushes could also possibly act as a bacterial reservior to contaminate patients’ oral cavity and cause lower air way infections. Another study examining the bacterial colonization status of toothbrushes of children with CF also found two identical *P. aeruginosa* clones between patient’s toothbrush and sputum samples and one patient’s sputum sample and his sibling’s toothbrush [11]. Our results are consistent with these studies with microscopy confirmation of bacterial biofilms presence as well.

Initial lung infections of children with CF can be cleared by vigorous antibiotic treatment. However, recurrent intermittent *P. aeruginosa* CF lung infection happens frequently [2], causing pulmonary inflammation and structural damage and leads to chronic infection [12]. The purpose of this study is focused on children with CF to reduce the frequency of recurrent infections and thus delay the onset of developing chronic lung infections. It is conceivable that infectious clones of *P. aeruginosa* and *S. aureus* may persist on toothbrushes as a biofilm. Following withdrawal of antibiotic treatment, the patient could possibly be reinfected from the biofilm on the toothbrush. Frequent recurrent lung infections cause irreversible lung damage and eventually results in chronic infection. The younger a patient develops chronic *P. aeruginosa* chronic infection, the more severe the disease and the greater the increased risk of death.

The limitation of this study is the small sample size due to funding restraints. Despite the small sample size, the detection of *P. aeruginosa* and *S. aureus* on toothbrushes of the children with CF, before receiving and during antibiotic treatment in this pilot study, demonstrated the potential of toothbrushes to act as reservoirs for recurrent lung infections. The other limitation is that LIVE/DEAD^®^ BacLight™ Bacterial Viability Kit used was only validated in certain species of planktonic bacteria, and it has not been validated in mixed species oral biofilm despite its wide use in biofilm studies [13]. Future studies need to investigate larger population size, collecting toothbrush samples from the same child before receiving, during and after antibiotic treatment. Future studies can also investigate other closely used personal items, such as toothbrush cups, face towels, pillowcases, toys, which may also act as potential reservoirs for recurrent infections.

We strongly recommend children with CF to replace their toothbrushes and thoroughly clean other closely touched personal items regularly and particularly after each acute infection episode and antibiotic treatment to avoid re-infection from these potential reservoirs, thus reducing infections from environmental sources and delaying the onset of chronic lung infection.

## Figures and Tables

**Table 1:** The number of *P. aeruginosa* and *S. aureus* cultured per bundle of toothbrush bristles, and bacterial species isolated from clinical and toothbrush samples.

Child ID	Bacterial Species in Sputum Sample	Toothbrush Collection Point	*P. aeruginosa* Number	*S. aureus* Number	Bacteria Species in Toothbrush Sample
1	*P. aeruginosa*	Before antibiotic treatment	4.87 × 10^5^ ± 4.03 × 10^4^	0	*P. aeruginosa* *Streptococcus mitis* *Enterobacter cloacae*
2	*S. aureus* *P. aeruginosa*	Before antibiotic treatment	2.76 × 10^5^ ± 3.96 × 10^4^	3.85 × 10^4^ ± 2.33 × 10^3^	*P. aeruginosa* *S. aureus* *Klebsiella oxytoca*
3	*P. aeruginosa* *Haemophilus influenzae*	Two weeks from starting antibiotic treatment	2.33 × 10^3^ ± 4.61 × 10^2^	0	*P. aeruginosa* *Micrococcus luteus* *Brachybacterium* spp.
4	*P. aeruginosa*	Three weeks from starting antibiotic treatment	3.64 × 10^3^ ± 5.07 × 10^2^	0	*P. aeruginosa* *S. epidermidis* *S. hominis* *Enterobacter cloacae* *Enterococcus faecium*
5	*S. aureus* *P. aeruginosa*	Three months after antibiotic treatment	0	0	*Kocuria* spp. *Streptococcus mutans*
6	*Stenotrophomonas maltophilia*	Seven months after antibiotic treatment	0	0	*Streptococcus sanguinis*

## Data Availability

The data generated are presented in this paper.
